# 1002. Activation of the *Enterococcus faecalis* Cell Envelope Stress Response through the Novel MadRS System Is Associated With Increased Size of Cardiac Microlesions

**DOI:** 10.1093/ofid/ofab466.1196

**Published:** 2021-12-04

**Authors:** William R Miller, William R Miller, Kavindra V Singh, Cesar A Arias

**Affiliations:** 1 Center for Antimicrobial Resistance and Microbial Genomics, UTHealth, Houston, TX; 2 Center for Antimicrobial Resistance and Microbial Genomics, UTHealth, Houston, TX, Houston, Texas; 3 CARMiG, UTHealth and Center for Infectious Diseases, UTHealth School of Public Health, HOU, TX ; Molecular Genetics and Antimicrobial Resistance Unit and International Center for Microbial Genomics, Universidad El Bosque, BOG, COL, Houston, Texas

## Abstract

**Background:**

Enterococci are opportunistic pathogens that can present a therapeutic challenge due to the acquisition of antibiotic resistance. Our previous work has shown the MadRS stress response system plays an important role in defending the enterococcal membrane against daptomycin and antimicrobial peptides (AMP) made by the innate immune system. Strains lacking the MadR response regulator show increased susceptibility to the cathelicidn LL-37 in vitro. A change from alanine to glutamate in the sensor kinase MadS (*madS*_*A202E*_) leads to activation of the system and impaired killing by AMPs. In this study, we evaluated the impact of MadRS function in vivo using a mouse peritonitis model of *E. faecalis* (*Efs*) infection.

**Methods:**

A laboratory strain *Efs* OG1RF and two derivatives, OG1RFΔ*madR* and OG1RF*madS*_*A202E*_ were included. Six mice per strain were inoculated via intraperitoneal injection of ~5x10^8^ CFU/mL of bacteria in 50% sterile rat fecal extract, and followed for 96 hours post infection. Difference in survival between strains was determined by Mantel-Cox test. At the time of death, hearts were aseptically removed, fixed in formalin, and embedded in paraffin. Organs were bisected and sectioned, with every 4^th^ section stained with hematoxylin and eosin (8 total sections per animal). Sections were imaged at 40x magnification, the number of lesions for each section was recorded, and lesion size was determined using imageJ.

**Results:**

There was no difference in median survival between animals infected with OG1RF and OG1RFΔ*madR* (22.5 v 21 hours, *p*=0.31), OG1RF and OG1RF*madS*_*A202E*_ (22.5 v 24 hours, *p*=0.29), or OG1RFΔ*madR* and OG1RF*madS*_*A202E*_ (21 v 24 hours, *p*=0.13). There was a significant difference in the number and size of cardiac lesions between the strains. Mice infected with OG1RF*madS*_*A202E*_ had a significantly higher number of cardiac microlesions as compared to those infected with OG1RFΔ*madR* (Fig 1). The size of the lesions in mice infected with OG1RF*madS*_*A202E*_ was also significantly larger than those in OG1RF wild type (Fig 1).

Figure 1. Cardiac microlesions in a mouse peritonitis model of Enterococcus faecalis infection.

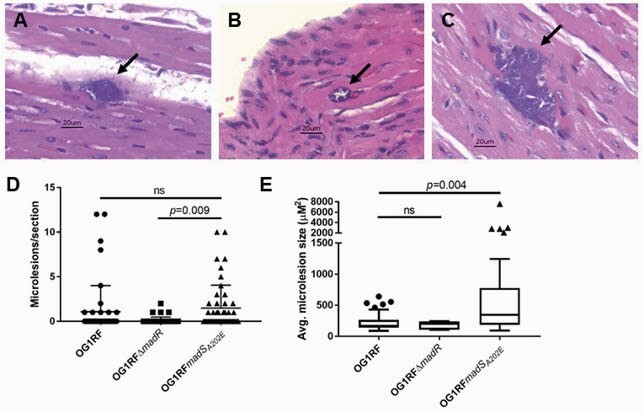

Mouse hearts were removed at time of animal death, placed in formalin, and embedded in paraffin. Organs were bisected, then sectioned with every 4th section stained with hematoxylin and eosin (H&E, 8 sections per animal). H&E stained sections were imaged at 40x magnification, the number and size of lesions was determined for 48 sections per strain. Representative cardiac microlesions (arrow) are shown for A) E. faecalis OG1RF, B) OG1RFΔmadR and C) OG1RFmadSA202E, scale bar 20 μm. D) The number of microlesions observed in each section, and E) the area of the lesions for each strain are shown above, differences in means were determined with one way ANOVA using Tukey’s test for multiple comparisons. ns, not significant.

**Conclusion:**

Changes in MadRS did not impact overall survival in mice, but did alter the number and size of cardiac microlesions. Further experiments are needed to determine if these changes could adversely affect therapy or rates of relapse.

**Disclosures:**

**William R. Miller, MD** , **Entasis Therapeutics** (Scientific Research Study Investigator)**Merck** (Grant/Research Support) **William R. Miller, MD** , Entasis (Individual(s) Involved: Self): Scientific Research Study Investigator; Merck (Individual(s) Involved: Self): Grant/Research Support **Cesar A. Arias, M.D., MSc, Ph.D., FIDSA**, **Entasis Therapeutics** (Grant/Research Support)**MeMed Diagnostics** (Grant/Research Support)**Merk** (Grant/Research Support)

